# Room Temperature Synthesis of Bioactive 1,2,4-Oxadiazoles †

**DOI:** 10.3390/ijms24065406

**Published:** 2023-03-12

**Authors:** Sergey V. Baykov, Anton A. Shetnev, Artem V. Semenov, Svetlana O. Baykova, Vadim P. Boyarskiy

**Affiliations:** 1 Institute of Chemistry, Saint Petersburg State University, 7/9 Universitetskaya Nab., 199034 Saint Petersburg, Russia; 2 Pharmaceutical Technology Transfer Centre, Yaroslavl State Pedagogical University Named after K.D. Ushinsky, 108 Respublikanskaya St., 150000 Yaroslavl, Russia

**Keywords:** 1,2,4-oxadiazole, room temperature, base catalyzed cyclization, oxidative cyclization, amidoximes, bioactive compounds, bioisosteres

## Abstract

1,2,4-Oxadiazole is an essential motif in drug discovery represented in many experimental, investigational, and marketed drugs. This review covers synthetic methods that allow the conversion of different types of organic compounds into 1,2,4-oxadiazole at ambient temperature and the practical application of the latter approaches for the preparation of pharmaceutically important molecules. The discussed methods are divided into three groups. The first combines two-stage protocols requiring the preliminary preparation of *O*-acylamidoximes followed by cyclization under the action of organic bases. The advantages of this route are its swiftness, high efficiency of the cyclization process, and uncomplicated work-up. However, it requires the preparation and isolation of *O*-acylamidoximes as a separate preliminary step. The second route is a one-pot synthesis of 1,2,4-oxadiazoles directly from amidoximes and various carboxyl derivatives or aldehydes in aprotic bipolar solvents (primarily DMSO) in the presence of inorganic bases. This recently proposed pathway proved to be highly efficient in the field of medicinal chemistry. The third group of methods consists of diverse oxidative cyclizations, and these reactions have found modest application in drug design thus far. It is noteworthy that the reviewed methods allow for obtaining 1,2,4-oxadiazoles with thermosensitive functions and expand the prospects of using the oxadiazole core as an amide- or ester-like linker in the design of bioactive compounds.

## 1. Introduction

1,2,4-Oxadiazole is an essential motif in drug discovery, which is incorporated in many experimental, investigational, and marketed drugs, for example, ataluren [1], naldemedine [2], amenamevir [3], ozanimod [4], azilsartan medoxomil [5], and opicapone [6]. Moreover, progress in the synthesis and medicinal application of the 1,2,4-oxadiazole core is being regularly reported (for recent reviews see Refs. [7,8,9,10,11,12]). The interesting feature of the 1,2,4-oxadiazole moiety from a medicinal chemistry viewpoint is its potential coverage of a broad spectrum of therapeutic areas, including oncology [13,14,15], immunology [16], neurology [17,18,19,20], infectious diseases [21,22,23,24], metabolism and endocrinology [25,26,27,28], urology [29,30], gastroenterology [31], cardiology [32], rheumatology [33], and respirology [34]. Such versatility of the 1,2,4-oxadiazole motif, in our opinion, is due to its recognition as the hydrolytically stable bioisostere of the amide and ester bonds [35,36,37,38,39]. There are many studies on the 1,2,4-oxadiazole ring acting as an amide-like linker with a suitable ADMET profile [40,41].

In addition, the popularity of 1,2,4-oxadiazoles in biomedical studies is, to a certain degree, associated with synthetic factors. The 1,2,4-oxadiazole core assembly consists of two stages (the *O*-acylation of amidoximes and the intramolecular cyclization [42]). The first is very similar to the amide bond formation in terms of starting reagents (acids and their derivatives) and conditions. Particularly, amidoximes have reactivity comparable to amines and can be acylated by a wide range of activated carboxylic acids (or their derivatives) at RT. In other words, the majority of methods utilized in the amide coupling [43] can also be employed for the synthesis of *O*-acylamidoximes, including the solid-support or combinatorial chemistry [44,45]. At the same time, the cyclization step commonly requires prolonged high-temperature (up to 140 °C) heating [42]). The necessity of thermal activation for the 1,2,4-oxadiazole ring closing is one of the main and the most disappointing limitations on the way to medicinal utilization of this heterocyclic motif. Thus, there was a need for new synthetic methods that allow the transformation of amidoximes to 1,2,4-oxadiazoles at ambient temperature. The first of these methods was developed by Gangloff, Rice, and colleagues in 2001 [46], and after a few years, it was in demand in the synthesis of potential pharmaceuticals [47].

Therefore, this review has two goals: on the one hand, to describe the progress and recent achievements in synthetic methodology in this field, and on the other hand, to consider issues related to the practical use of this approach for the preparation of biologically active 1,2,4-oxadiazoles.

## 2. Base-Induced Cyclodehydration of *O*-acylamidoximes

### 2.1. Tetrabutylammonium Fluoride (TBAF)

**Scheme 1:**
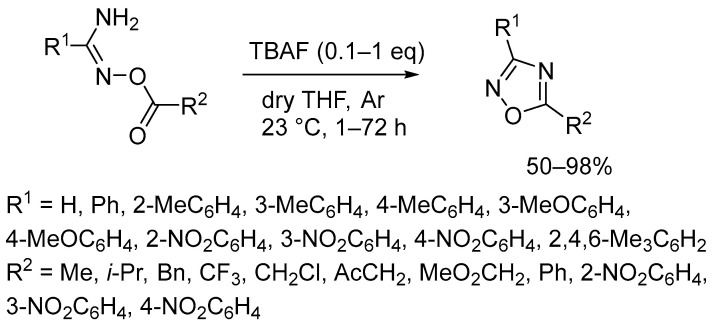
Room-temperature cyclization of *O*–acylamidoximes under TBAF catalysis.

As mentioned above, the first room-temperature synthesis of 3,5-disubstituted 1,2,4-oxadiazoles was reported by Gangloff, Rice, and colleagues in 2001. Upon treatment with TBAF (in stoichiometric or catalytic amounts), *O*-acylamidoximes underwent mild and facile conversion into 1,2,4-oxadiazoles in THF at room temperature (Scheme 1) [46].

**Scheme 2:**
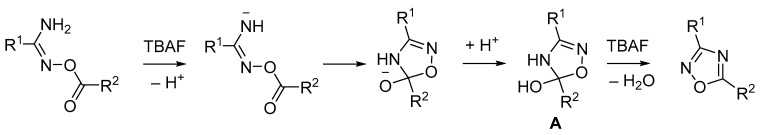
Mechanism of TBAF-catalyzed synthesis of 1,2,4-oxadiazoles.

TBAF functions as a strong base in dry THF [48,49]. The reaction proceeds via intermediate **A** formation (Scheme 2). This intermediate can be isolated by flash chromatography if the reaction is quenched at 0 °C. The authors demonstrated that the intermediate **A** is stable in solution by itself, but rapidly dehydrates under the action of TBAF.

The same research group used this approach for the 1,2,4-oxadiazole synthesis on a solid support [50].

**Scheme 3:**
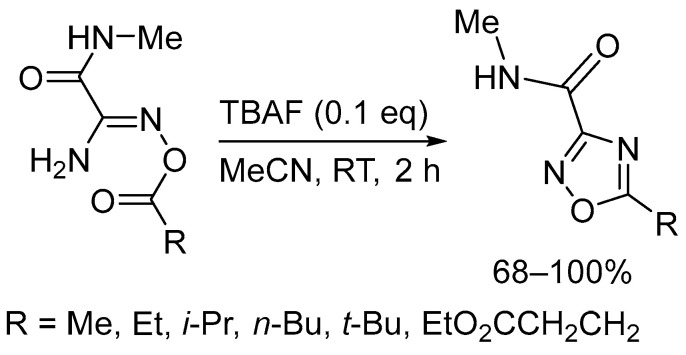
Synthesis of carbamoyloxadiazoles under TBAF catalysis.

Nishiwaki, Ariga et al. described the synthesis of carbamoyloxadiazoles from *O*-acylamidoximes (Scheme 3) [51]. The authors compared several protocols for the 1,2,4-oxadiazole core formation and found that the addition of a catalytic amount of TBAF in an acetonitrile solution gave the best results. The *O*-alkanoylamidoximes were transformed into the corresponding oxadiazoles in either quantitative (R = Alk) or good (R = EtO_2_CCH_2_CH_2_) yields at room temperature even though bulky alkanoyl groups were employed. However, in cases of aroylamidoximes, corresponding oxadiazoles could be prepared effectively only by heating at 50–80 °C.

**Scheme 4:**
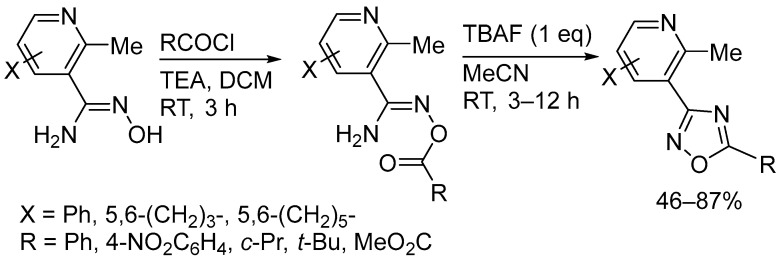
Synthesis of sterically hindered 3-(1,2,4-oxadiazol-3-yl)pyridines under TBAF catalysis.

Lukyanov et al. described the synthesis of sterically hindered 3-(1,2,4-oxadiazol-3-yl)pyridines via a two-step process from substituted *N*-hydroxy-pyridine-3-carboximidamides and acyl chlorides using the room-temperature cyclization of *O*-acylamidoximes with TBAF in MeCN as the second step (Scheme 4) [52]. Note that *O*-aroylamidoximes cyclized as well as *O*-alkanoylamidoximes.

**Scheme 5:**
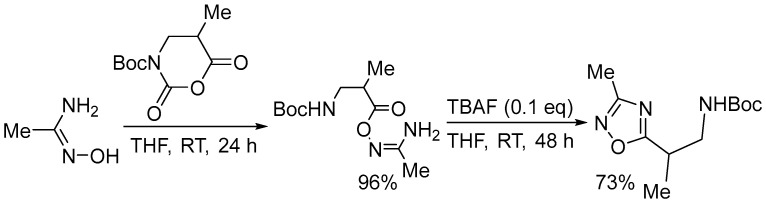
*N*-Boc-*α*-methyl-*β*-alanine-*N*-carboxyanhydride as an acylating agent.

The mild reaction conditions (room temperature and the absence of hydrolyzing agents) allowed Huck and colleagues to use this method in the synthesis of the oxadiazole-bearing Boc-protected amino group from acetyl amidoxime and *N*-Boc-*α*-methyl-*β*-alanine-*N*-carboxyanhydride (Scheme 5) [53]. For the synthesis of analogous derivatives from other amidoximes RC(NH_2_)OH (R = Ph, *t*-BuO_2_CCH_2_, CH_2_C(NH_2_)OH), the authors suggested adding to the reaction mixture 1 eq of triethylamine (TEA) alongside TBAF. Then, the process can be carried out without isolating *O*-acylamidoxime intermediates.

The experimental procedure proposed by Gangloff, Rice, and colleagues [46] has not changed significantly since then, and it has been used “as is” in the laboratory practice for the preparation of many pharmaceutically relevant scaffolds containing the 1,2,4-oxadiazole ring. We found approximately thirty works related to this topic, and in the context of this review, it is redundant to discuss the synthesis of each scaffold in detail. The general experimental procedure is the *O*-acylation of an amidoxime with an acyl chloride, an anhydride, or an activated carboxylic acid (most commonly) in a suitable solvent, followed by cyclocondensation of the *O*-acylamidoxime to 1,2,4-oxadiazole in the presence of TBAF/THF at RT. The cyclocondensation time correlates with the amount of TBAF (0.1–1.4 eq) and typically ranges from 1 to 12–16 h (overnight). It is important that in almost all cases it was necessary to isolate the intermediate *O*-acylamidoximes. Table 1 illustrates the diversity and complexity of biologically active scaffolds available through TBAF. There are anticancer, antimicrobial, and antiviral agents, neuroprotectors, immunomodulators, as well as metabolic regulators.

**Scheme 6:**
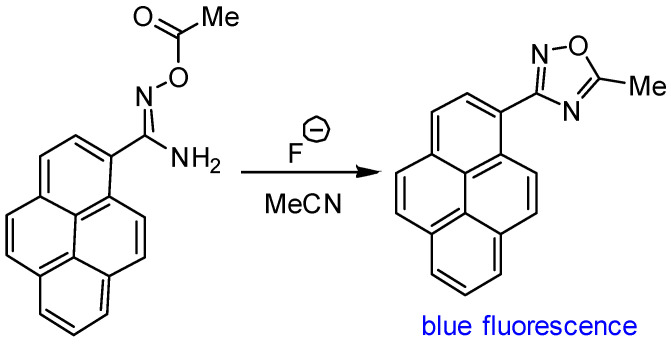
Sensing of fluoride based on the *O*-acylamidoxime cyclocondensation.

In addition to its extensive medicinal applications, the fluoride-induced cyclocondensation of *O*-acylamidoximes into 1,2,4-oxadiazoles at room temperature was used for the design of anion receptors [80]. Malkondu et al. synthesized non-light-emitted pyrene-substituted *O*-acylamidoxime, which closed into the corresponding 1,2,4-oxadiazole with blue-light fluorescence in the presence of fluoride (Scheme 6). This feature allows selective recognition of fluoride among a wide of range other anions, including chloride, bromide, acetate, nitrate, perchlorate, and hydrogen sulfate, as well as dihydrogen phosphate. 

**Scheme 7:**
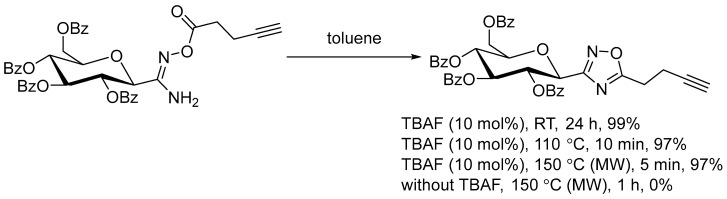
Synthesis of glycosylated 1,2,4-oxadiazole via TBAF-catalyzed cyclization.

Vidal with coworkers reported that TBAF catalysis can be combined with thermal activation [81]. Particularly, they investigated the cyclodehydration of the carbohydrate-substituted *O*-acylamidoxime into the corresponding 1,2,4-oxadiazole in the presence of TBAF at RT, 110 °C (conventional heating), and 150 °C (MW) (Scheme 7). Although the thermal activation drastically decreased the reaction time, the same product yield (97–99%) was achieved in all conditions. Notably, the target 1,2,4-oxadiazole was not formed in the absence of the catalyst. 

In conclusion, the TBAF-mediated approach [46] demonstrates a very high efficiency for the overall cyclization of *O*-acylamidoximes to 1,2,4-oxadiazoles, including a wide range of heterocycles with different functional groups. This process is highly productive, and the work-up procedure is relatively simple. The reactions are carried out under mild conditions at temperatures from 0 °C to room temperature. However, the use of *O*-acylamidoximes as starting materials is associated with additional labor costs for their preparation and isolation, which complicates the synthesis and reduces the overall yield of 1,2,4-oxadiazoles.

### 2.2. Tetrabutylammonium Hydroxide (TBAH)

**Scheme 8:**
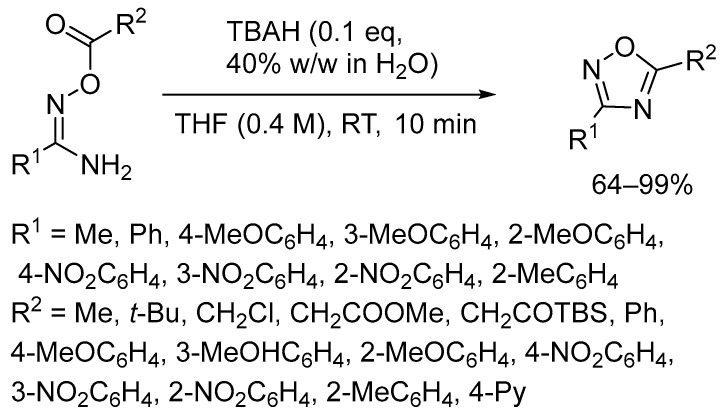
TBAH-catalyzed *O*-acylamidoxime cyclization.

In 2014, Otaka with coworkers from Dainippon Sumitomo Pharma Co., Ltd. (Osaka, Japan) suggested an alternative to the TBAF-base catalyst for the cyclodehydration of *O*-acylamidoximes into 1,2,4-oxadiazoles [82]. During the drug discovery process, the authors faced difficulties with the large-scale synthesis of 1,2,4-oxadiazoles, which were caused by the high corrosive action of the fluorine anion on conventional reaction vessels. To solve this problem, they performed a study for researching an alternative catalyst that would be more suitable for production scale. A wide range of compounds including tetrabutylammonium derivatives with different anions (chloride, bromide, cyanide, hydrogen sulfate, and hydroxide), TEA, and DBU was examined, and tetrabutylammonium hydroxide (TBAH) was recognized as the most effective (Scheme 8). Other tetraalkylammonium hydroxides (Pr_4_NOH, Et_4_NOH, and Me_4_NOH) also displayed good catalytic activity. Moreover, a moderate product yield (65%) was obtained using NaOH as a hydroxide source. In addition to the base nature, the authors also studied the solvent effect on cyclodehydration. The reaction in most aprotic solvents (DMF, THF, DCM, MeCN, and acetone) and nonprimary alcohols (*i*-PrOH and *t*-BuOH) resulted in excellent (88–95%) isolated yields of 1,2,4-oxadiazole, whereas toluene (trace), ethyl acetate (30%), water (trace), MeOH (0%), and EtOH (19%) were found to be unsuitable for this transformation.

The reaction scope investigation revealed some advantages of TBAH in comparison with TBAF: the shorter reaction time (10 min instead 1 h) and the compatibility with silyl-protecting groups (for example, TBS). Nevertheless, despite this being known for the 8 past years, we found only one mention of 1,2,4-oxadiazole core synthesis in the presence of TBAH [83]. Maybe it is due to the lack of awareness or some conservatism of the synthetic community. The lesser availability of TBAH in comparison with TBAF, perhaps, also has a contribution.

### 2.3. Alkali Metal Bases

**Scheme 9:**
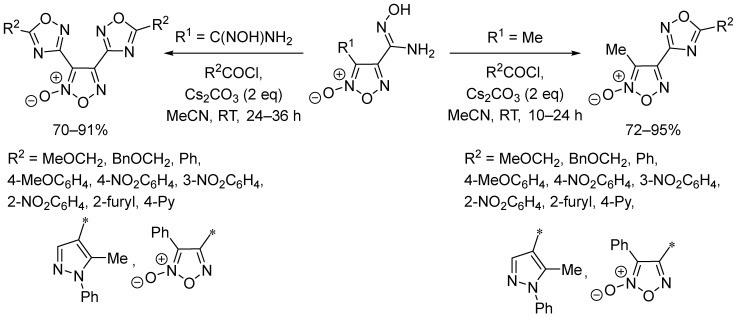
Preparation of various 1,2,4-oxadiazol-furoxan hybrids.

To the best of our knowledge, the first example of the inorganic base-induced 1,2,4-oxadiazole ring formation at room temperature was reported by Fershtat et al. [84]. They developed the protocol for the synthesis of 1,2,4-oxadiazol/1,2,5-oxadiazole 2-oxide hybrids via the cyclocondensation of furoxanylamidoximes with acyl chlorides in the presence of Cs_2_CO_3_ in MeCN at 20 °C for 10–36 h (Scheme 9). The other important feature of this work is that it is also the first example of the one-pot room temperature procedure: all 22 products were obtained in good to excellent yields without the *O*-acylamidoxime isolation. The structural diversity of products mostly was provided by the varying acyl chlorides, whereas the amidoxime scope was represented by only two 1,2,5-oxadiazole 2-oxide derivatives. The cytotoxic effect of several synthesized hybrids against human cancer cell lines was evaluated, but the tested compounds did not demonstrate any significant activity [85].

We did not reveal any further evolution of this method.

**Scheme 10:**
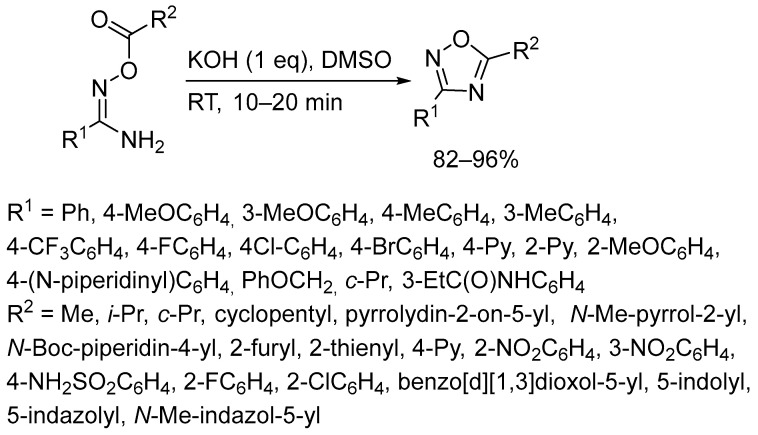
Cyclization of *O*–acylamidoximes into 1,2,4-oxadiazoles in KOH/DMSO medium.

In 2016, Baykov et al. studied cyclocondensation of *O*-benzoylbenzamidoxime in the presence of a wide range of bases, including alkali metal hydroxides (LiOH, NaOH, and KOH), carbonates, and bicarbonates (Na_2_CO_3_, K_2_CO_3_, Cs_2_CO_3_, NaHCO_3_, and KHCO_3_), as well as sodium acetate and TEA in DMSO at RT (Scheme 10) [86]. It was found that all hydroxides demonstrated comparable activity and provided 3,5-diphenyl-1,2,4-oxadiazole in a good yield of about 98% for 10 min. Additionally, slightly lower product yields were obtained with Cs_2_CO_3_ (86%) and K_2_CO_3_ (71%), whereas only a trace amount of the 1,2,4-oxadiazole was detected in cases of other bases. In addition, the catalytic effect of the base was proved by the reaction with 0.1 eq of KOH. To examine the reaction scope, the authors synthesized 22 1,2,4-oxadiazoles bearing diverse functionalities in KOH/DMSO medium.

**Scheme 11:**
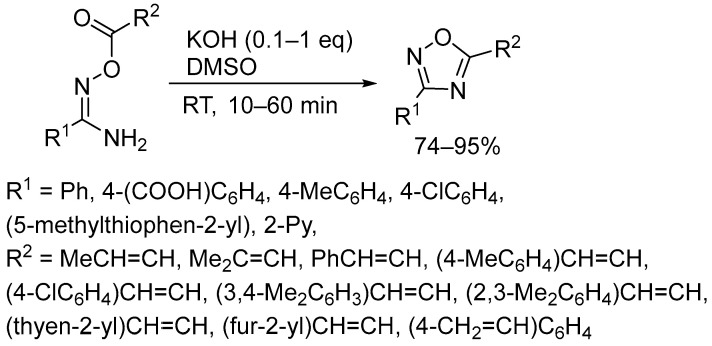
Cyclization of *O*–acylamidoximes with double bond in KOH/DMSO medium.

In the next work of the same group, the cyclocondensation of *O*–acylamidoximes bearing carbon–carbon double bonds in KOH/DMSO medium was investigated (Scheme 11) [87]. It was found that the result of the reaction strongly depends on the type and position of a double bond. Particularly, 1,2,4-oxadiazoles with the terminal double bond attached directly to the heterocyclic core (R^2^ = CH_2_=CH, CH_2_=C(Me)) were obtained in very poor yields (11% and 15% correspondingly) only, presumably, due to the KOH-initiated anionic polymerization. Derivatives of crotonic and methyl crotonic acids, also, turned out to be sensitive to basic conditions; however, corresponding 1,2,4-oxadiazoles were isolated in good yields by using 0.1 eq of KOH. At the same time, derivatives of cinnamic and 4-vinylbenzoic acids were synthesized mostly in a good yield according to the general conditions without any difficulties. For example, Vasilyev et al. reported the triflic acid-catalyzed hydroarylation of the carbon–carbon double bond in 5-(2-arylethenyl)-3-aryl-1,2,4-oxadiazoles, which were prepared preliminarily under KOH/DMSO conditions [88].

**Scheme 12:**
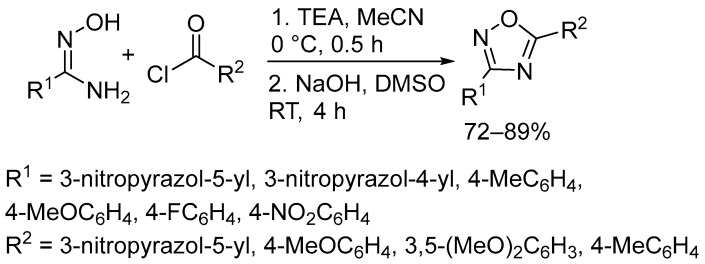
Synthesis of 1,2,4-oxadiazolones bearing nitro-groups.

Moreover, MOH/DMSO (M = Na or K) system was found to be an essential medium for synthesis of 1,2,4-oxadiazoles containing nitro-groups [89,90]. Particularly, Dalinger et al. demonstrated that *O*-acylamidoximes substituted by 3-nitro-pyrazole moiety closed into corresponding oxadiazoles in the NaOH/DMSO system for 4 h in a good yield (Scheme 12), whereas prolonged (over 24 h) refluxing in the presence of TEA in MeCN was unsuccessful [89].

**Scheme 13:**
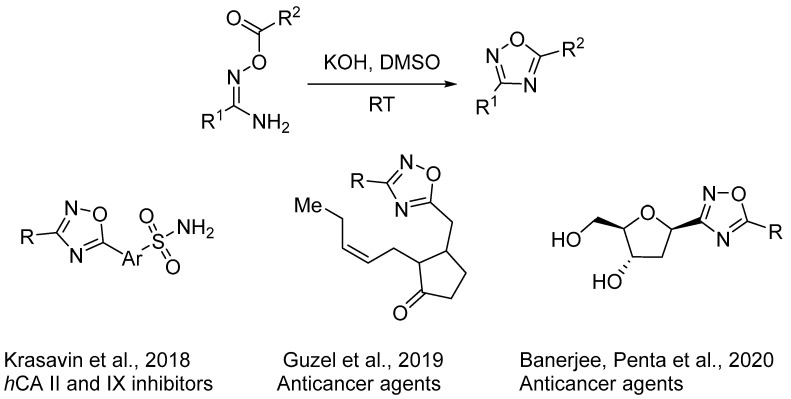
Cyclization of *O*-acylamidoximes into bioactive 1,2,4-oxadiazoles in KOH/DMSO medium [91,92,93].

The cyclocondensation of *O*–acylamidoximes in KOH/DMSO medium was mentioned in several medicinal chemistry studies to date (Scheme 13) [91,92,93,94]. To discover new potent and selective human carbonic anhydrase (*h*CA) inhibitors, Krasavin et al. prepared a series of 1,2,4-oxadiazoles bearing primary arylsulfonamide at position 5 of the heterocyclic core [91]. Obtained compounds demonstrated significant activity toward cytosolic *h*CA II and membrane-bound *h*CA IX isoforms. Guzel et al. obtained three methyl jasmonate derivatives with 1,2,4-oxadiazole motif as hexokinase-II inhibitors for anticancer therapy [92] The therapeutic potential of these heterocycles was proven by the enzyme inhibition assay followed by cytotoxicity experiments on A549 and SKOV-3 cell lines. Notably, exactly the 1,2,4-oxadiazole core was found most active among the considered heterocyclic motifs (isomeric 1,3,4-oxadiazole, isoxazole, and thiazole). To the same purpose, 2′-deoxy-*C*-nucleosides decorated by a 1,2,4-oxadiazole periphery were synthesized and evaluated toward five tumor cell lines, namely, HeLa, MDAMB-231 (breast cancer), PANC-1 (pancreatic cancer), PC3 (prostate cancer), and SK-OV-3 (ovarian cancer) [93]. Finally, Tu et al. used the KOH/DMSO medium in the development of fluorine-18 radiotracer [^18^F]FS1P1 for imaging sphingosine-1-phosphate receptor 1 [94].

As mentioned above, the cyclocondensation of *O*-acylamidoximes is a not so attractive method, and one-pot protocols, which start from amidoximes, are much more desirable, primarily, for drug discovery. The possibility of carrying out the reaction in a “two-step, one-pot” manner is the main advantage of the MOH/DMSO system (M = Li, Na, K) in comparison with TBAF-involved methods, and it was realized in several works [95,96,97,98,99,100,101,102,103]. It is most convenient to discuss these methods by dividing them by the type of used carboxyl function sources: esters, acids, acyl chlorides, anhydrides, dicarboxylic acid anhydrides, aldehydes, and even *N*-acyl benzotriazoles. 

**Scheme 14:**
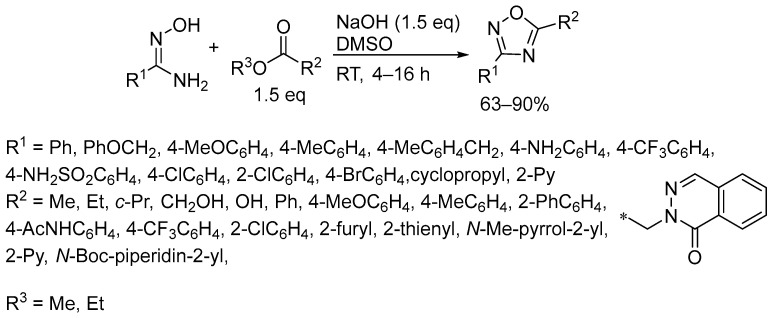
Reaction of amidoximes with esters in NaOH/DMSO medium.

Esters as the carboxyl function source are the most favorable reagents for the 1,2,4-oxadiazole core assembly in basic conditions. In this case, a base promoted both *O*-acylation and intramolecular cyclocondensation stages and any special additives usually are not required. It was demonstrated that the condensation of esters and amidoximes into 1,2,4-oxadiazoles occurs in the MOH/DMSO medium (M = Li, Na, K) at RT and typically takes from 4 to 16 h depending on the reagent structure (Scheme 14) [95]. The optimization of reaction conditions revealed NaOH as the most suitable base for this process. 

The reaction scope includes a broad spectrum of alkyl, aryl, and hetaryl amidoximes and esters, as well as many functional groups; nevertheless, some limitations were recognized. Namely, derivatives of NH-heterocycles (R^2^ = pyrrole-2-yl, indole-5-yl, piperidine-4-yl) gave significantly lower yield (11%, 14%, and 30%, respectively). In addition, aryl esters bearing strong electron-donating substituents such as hydroxyl or amino groups at the *para*-position of the aromatic ring are not reactive in these conditions. Later, Yun Young Go et al. [104] and Kinzhalov et al. [105] showed that mild heating (30–60 °C) led to obtaining 1,2,4-oxadiazoles with unprotected primary arylamino functionalities. At the same time, Teleb with colleagues successfully performed the reaction with salicylic esters at RT [106]. One more limitation is the presence of a high CH-acidic methylene group. Particularly, the acetoacetic ester and methyl 2-(*p*-tolyl)acetate did not afford the desired heterocycles. Interestingly, ethyl 2-(1-oxophthalazin-2(*1H*)-yl)acetate provided the corresponding 1,2,4-oxadiazole with a yield of 79%. 

**Scheme 15:**
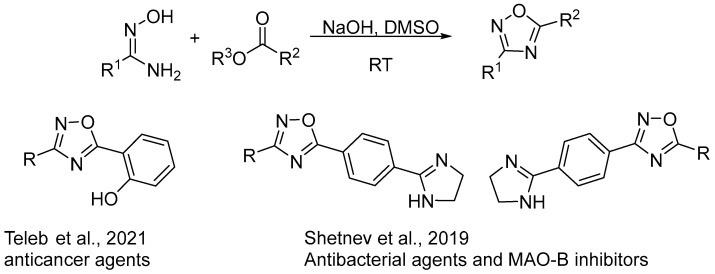
Preparation of bioactive 1,2,4-oxadiazoles from esters and amidoximes [106,109,110].

The synthetic capabilities of this reaction in areas of organic and medicinal chemistry were explored by several research groups (Scheme 15) [106,107,108,109,110]. The abovementioned Teleb’s work [106] was dedicated to preventing oxidative stress and carcinogenesis via the indirect activation of the nuclear factor erythroid 2 p45-related factor 2 (Nrf2). For this goal, the authors investigated the inhibition of Nrf2 suppressors: thioredoxin reductase 1 (TrxR1), inhibitory kappa B kinase α (IKKα), and nuclear factor kappa B (NF-kB) by 1,2,4-oxadiazole derivatives. It is noteworthy that according to the results of their enzyme and cell experiments, the most potent compounds in the series were 2-substituted phenols. Novikov et al. synthesized a number of 1,2,4-oxadiazoles as starting materials for the transformation into 5-sulfonamidoimidazoles [107] or 2*H*-1,3,5-oxadiazines [108] via metal-carbenoid-induced ring cleavage. Shetnev et al. obtained 1,2,4-oxadiazole/2-imidazoline hybrids and demonstrated their prospect for the treatment of bacterial infections and pancreatic ductal adenocarcinoma [109]. Moreover, the same scaffold showed a significant inhibitory action toward monoamine oxidase B (MAO-B) in combination with good monoamine oxidase A (MAO-A) selectivity [110]. 

**Scheme 16:**
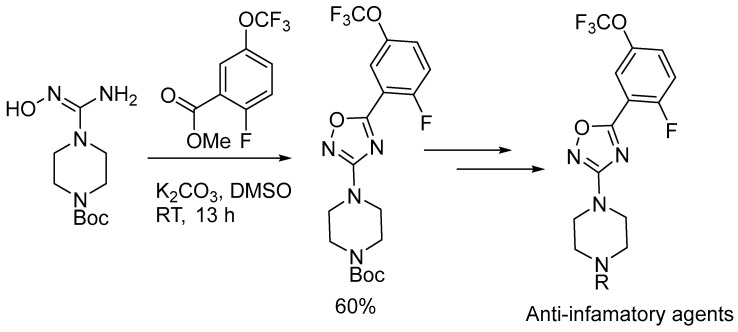
Synthesis of anti-inflammation agents based on the 1,2,4-oxadiazol-3-yl)piperazine scaffold.

There are a few examples of carrying out this reaction with other bases. Manjunatha et al. designed and synthesized a series of 1,2,4-oxadiazol-3-yl)piperazines with anti-inflammatory activity (Scheme 16). The heterocyclic core assembly was performed in DMSO in the presence of powdered K_2_CO_3_ for 13 h [111].

**Scheme 17:**
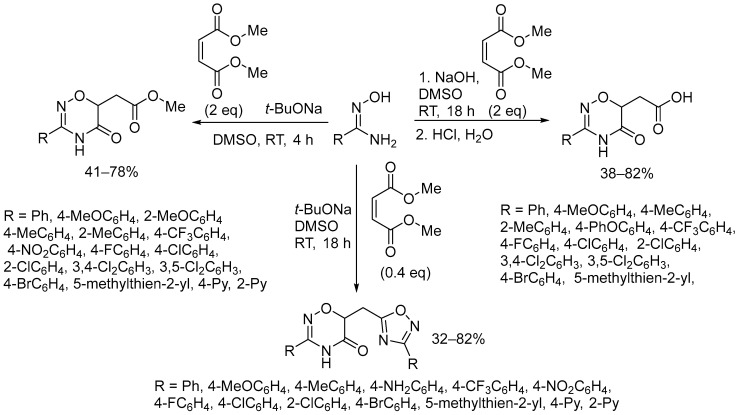
Synthesis of 1,2,4-oxadiazin-5(6*H*)-ones from amidoximes and maleic dimethyl ester.

Recently, Boyarskiy with coworkers reported that the reaction between amidoximes and maleic esters in NaOH/DMSO medium provided 1,2,4-oxadiazin-5(6*H*)-ones instead of the expected 1,2,4-oxadiazoles (Scheme 17). The base replacement to *t*-BuONa and increasing the amidoxime amount up to 2.5 eq allowed them to obtain hybrid 3-(aryl)-6-((3-(aryl)-1,2,4-oxadiazol-5-yl)methyl)-4*H*-1,2,4-oxadiazin-5(6*H*)-ones [112]. 

**Scheme 18:**
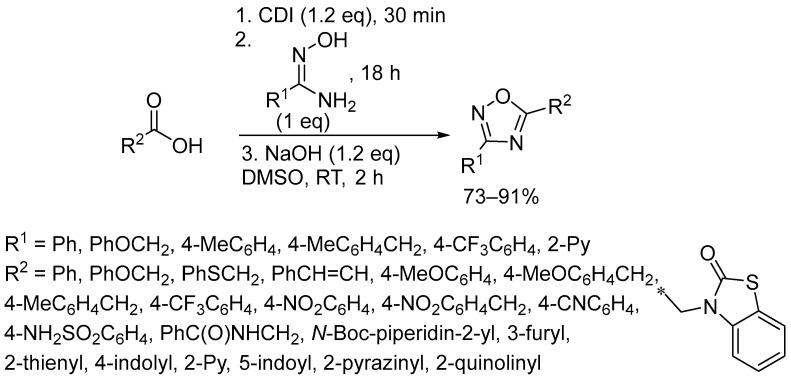
Reaction of amidoximes and carboxylic acids in NaOH/DMSO medium.

Carbonyldiimidazole (CDI) was found to be the appropriate activating agent for the synthesis of 1,2,4-oxadiazoles from amidoximes and carboxylic acids in the NaOH/DMSO medium [97]. Ethyl and isobutyl chloroformates in combination with organic bases (TEA or pyridine) also can be used instead of CDI; however, the yield of 1,2,4-oxadiazoles will be less. The reaction scope was demonstrated by 26 different examples (Scheme 18). This protocol was used by Arifuddin et al. for the preparation of coumarins bearing the 1,2,4-oxadiazole moiety as selective inhibitors of cancer-related *h*CA IX and XII isoforms [113].

Since “esters” and “acids” routes complement each other, allowing wider variation of initial reagents (esters or acids), in some works both routes are applied [14,114,115]. For example, Boyarskiy et al. reported the successful synthesis of 1,2,4-oxadiazoles substituted by pyridine *N*-oxides [115], and the *N*-oxide functionality was compatible with both “ester” and “acid” protocols. Furthermore, these *N*-oxides were converted into corresponding *N*-pyridyl ureas [115,116], which are valuable “masked isocyanates” [117,118] and ligands of metal-based anticancer drugs [119,120]. Krasavin et al. continued the exploration of 1,2,4-oxadiazole motif for the design of carbonic anhydrase inhibitors [14,114]. They discovered a number of compounds with the sub-nanomolar inhibition level against *h*CA IX isoform. The selected hits selectively kill pancreatic cancer (PANC-1) and melanoma (SK-MEL-2) cells in the hypoxic environment.

**Scheme 19:**
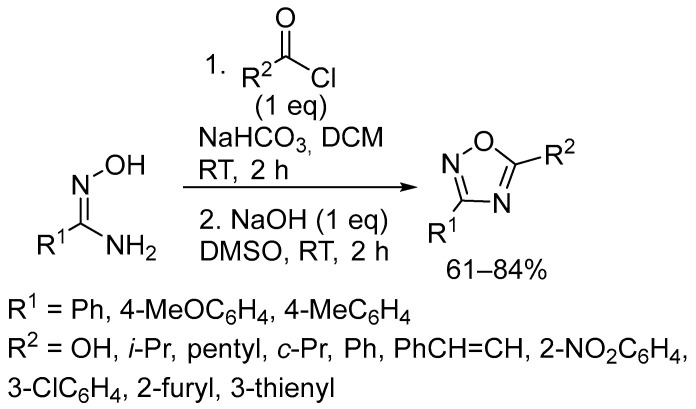
Reaction of amidoximes with acyl chlorides in NaOH/DMSO medium.

The reaction of amidoximes with acyl chlorides and anhydrides in the NaOH/DMSO medium directly provided only moderate yields of 1,2,4-oxadiazoles [96]; however, the yield can be improved by using other solvents (for example, DCM) for the acylation step (Scheme 19). Although in this case, the more complicated work-up associated with the isolation of *O*–acylamidoxime is required, the latter does not require additional purification before the cyclocondensation step. This technique was utilized by Postnikov and coauthors to create structural diversity of 3-substituted 1,2,4-oxadiazolones [121].

The synthesis of 1,2,4-oxadiazole bearing carboxylic functionality via the one-pot condensation of amidoximes and dicarboxylic acid anhydrides is one of the major benefits from the exploitation of the MOH/DMSO medium [98,99,100,101,102]. Notably, successful examples of the base-catalyzed condensation of *O*-acylamidoximes containing carboxylic functionality are not described. Moreover, the inapplicability of TBAF for this purpose was demonstrated by Nishiwaki with colleagues [51]. 

**Scheme 20:**
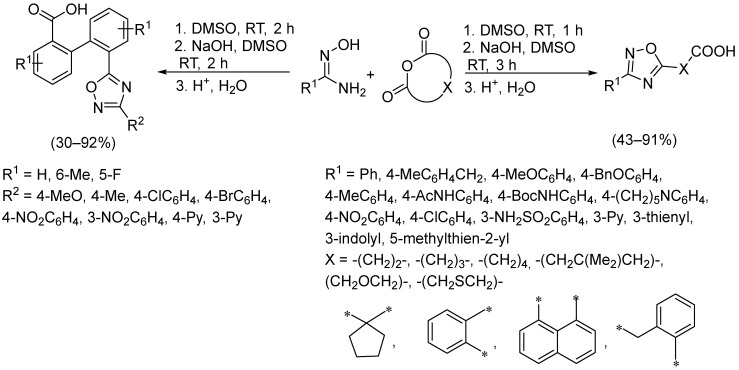
Reaction of amidoximes with dicarboxylic acid anhydrides in NaOH/DMSO medium.

The general reaction conditions include the treatment of amidoximes with an anhydride in DMSO for 1–18 h (depending on the anhydride reactivity) followed by the addition of NaOH to perform cyclocondensation (~3 h) (Scheme 20). The method allows obtaining 1,2,4-oxadiazole derivatives of succinic, glutaric, adipic, phthalic, and homophthalic anhydrides in moderate to excellent yields. Several derivatives of biphenylcarboxylic acid prepared according to this procedure have shown modest antibacterial action against *Escherichia coli* [102].

**Scheme 21:**
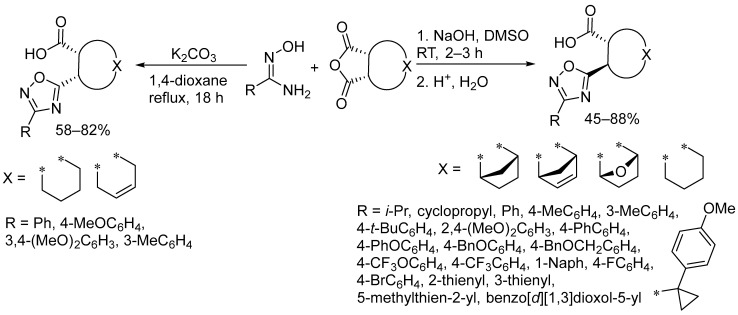
Synthesis of cycloalkyl carboxylic acids bearing 1,2,4-oxadiazole moiety.

Moreover, previously undescribed 1,2,4-oxadiazole/norborna(e)ne hybrids were synthesized from norbornene and norbornane dicarboxylic acids anhydrides according to this procedure (Scheme 21) [99]. The feature of this reaction is an epimerization and the mentioned 1,2,4-oxadiazole/norborna(e)ne hybrids were obtained as *trans*-diastereomers, whereas the starting anhydrides had *cis*-conformation. The same inversion was also observed for the reaction of amidoximes with anhydrides of cyclohexane- and cyclohexene-1,2-dicarboxylic acids [100]. Although the detailed mechanism of epimerization is not clear hitherto, strong basic conditions are of key importance. For example, the same reaction in the presence of K_2_CO_3_ in 1,4-dioxane provides *cis*-isomers (Scheme 21) [99,100].

**Scheme 22:**
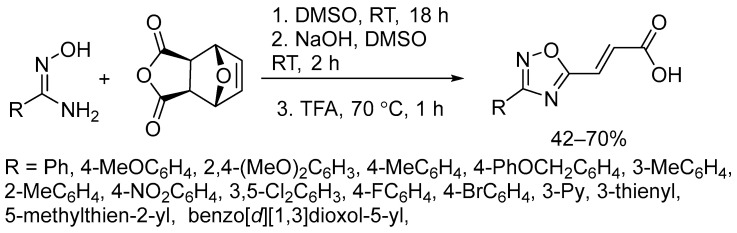
Synthesis of 1,2,4-oxadiazole substituted acrylic acids.

Unfortunately, these conditions are not suitable for maleic anhydride, probably due to the carbon–carbon double bond. However, the corresponding 1,2,4-oxadiazole substituted acrylic acids were synthesized via the ring-opening/ring-closing/retro-Diels-Alder reaction sequence from amidoximes and exo-3,6-epoxy-1,2,3,6-tetrahydrophthalic anhydride in DMSO medium (Scheme 22) [101]. As in the cases of other bridged anhydrides, the *cis*- to *trans*-epimerization occurred and the isolated products have *E*-conformation.

**Scheme 23:**
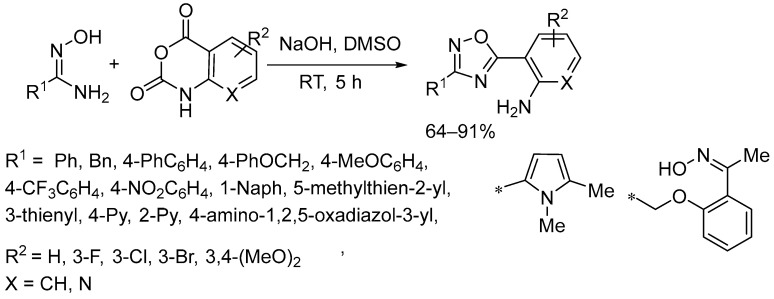
Synthesis of 2-(1,2,4-oxadiazol-5-yl)anilines.

In addition, after a minor optimization, the method was expanded on isatoic anhydrides [122] and used for the preparation of the library of 2-(1,2,4-oxadiazol-5-yl)anilines (Scheme 23), which were recognized as a new scaffold for blue-light-emitted materials [123].

**Scheme 24:**
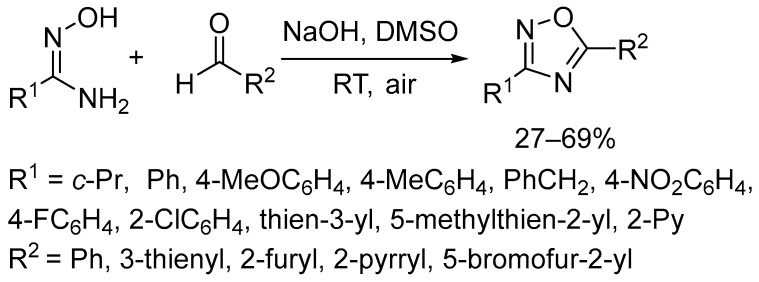
Reaction of amidoximes with aldehydes in NaOH/DMSO medium.

It was shown that the reaction of aromatic and heteroaromatic aldehydes with amidoximes in NaOH/DMSO medium in air (open flask) leads to 3,5-disubstituted-1,2,4-oxadiazoles (Scheme 24) [103].

Ott et al. reported the formation of the 1,2,4-oxadiazole core via the condensation of amidoximes with *N*-acyl benzotriazoles in *t*-BuOK/DMSO system at RT [124].

**Scheme 25:**
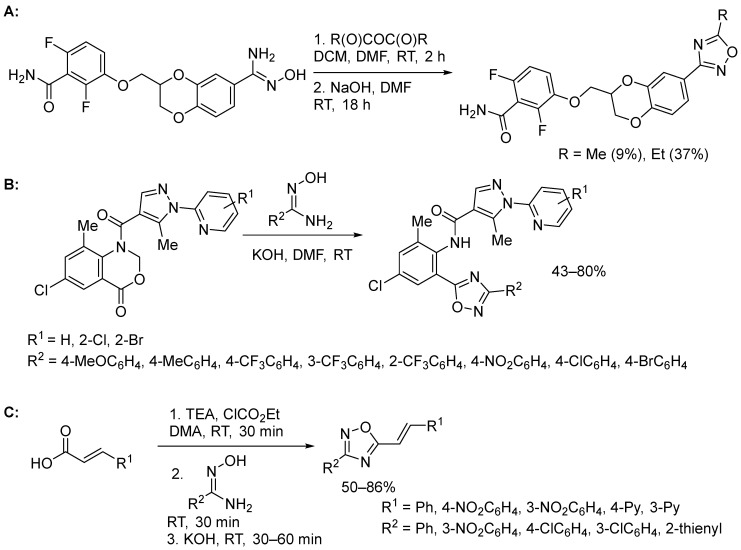
Synthesis of 1,2,4-oxadiazoles in aprotic solvents other than DMSO: in NaOH/DMF medium (**A**); in KOH/DMF medium (**B**); in KOH/DMA medium (**C**).

Finally, a few reports indicated that DMSO can be replaced by DMF or DMA [125,126,127,128]. Straniero et al. obtained two new 1,2,4-oxadiazole-based inhibitors of filamentous temperature-sensitive protein Z (FtsZ) for the treatment of drug-resistant bacterial infections (Scheme 25A) [125]. The synthesis concluded in the one-pot reaction of the starting benzodioxane-substituted amidoxime with acetic or propionic anhydrides followed by the NaOH-catalyzed cyclocondensation of *O*-acylamidoxime intermediates at RT for 18 h. Disappointingly, despite the prolonged reaction time, product yields were only 9% and 37% for acetic and propionic derivatives, respectively. Zhu et al. described the room temperature condensation of *4H*-3,1-benzoxazinones with amidoximes in KOH/DMF medium (Scheme 25B) [126]. The resulting anthranilic diamides bearing the incorporated 1,2,4-oxadiazole moiety were obtained in moderate to good yields. Recently, Sidneva et al. developed the one-pot protocol for the synthesis of 1,2,4-oxadiazole at room temperature via the reaction of cinnamic acids activated by ethyl chloroformate with amidoximes in the presence of KOH in dimethylacetamide (DMA) (Scheme 25C) [127]. In this case, yields of final heterocycles were comparable with previously reported data for reactions in DMSO. Another recent example of successful 1,2,4-oxadiazole synthesis in DMA is the *t*-BuONa-promoted one-pot condensation between esters bearing the primary sulfonamide functionality and amidoximes reported by Shetnev with coworkers [128].

## 3. Oxidative Cyclization

More recently, a new approach to the low-temperature synthesis of 1,2,4-oxadiazoles has emerged, which can be called oxidative cyclization. In this case, the heterocyclic core is formed due to the oxidative coupling of the N–H and O–H or C–H bonds. 

**Scheme 26:**
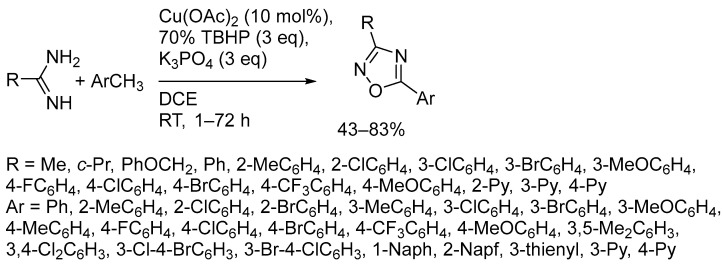
Copper-catalyzed cascade oxidative reaction of amidines and methylarenes.

Jiang and colleagues reported obtaining a wide range of 3,5-disubstituted-1,2,4-oxadiazoles in moderate to good yields from amidines and methylarenes using a one-step copper-catalyzed cascade reaction under mild conditions (Scheme 26) [129].

**Scheme 27:**
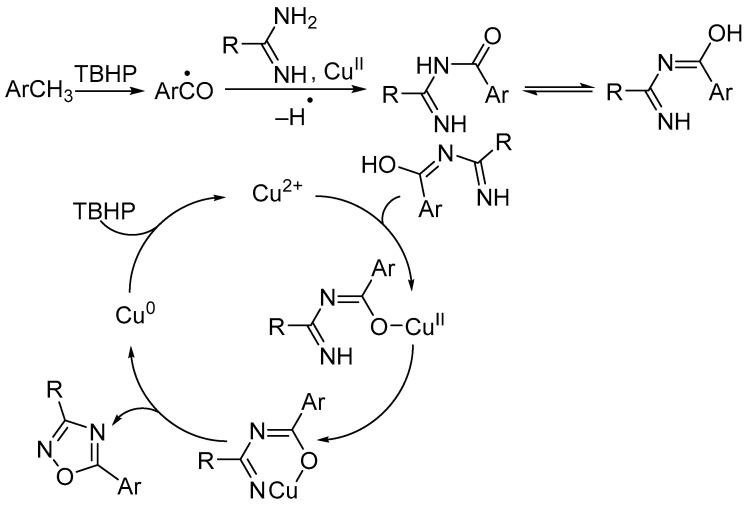
Mechanism of the copper-catalyzed oxidative process.

The reaction is a one-pot oxidation–amination–cyclization tandem process and involves radical C–H bond oxidation by TBHP and copper-catalyzed oxidative C–N/C–O/N–O bonds formation (Scheme 27).

**Scheme 28:**
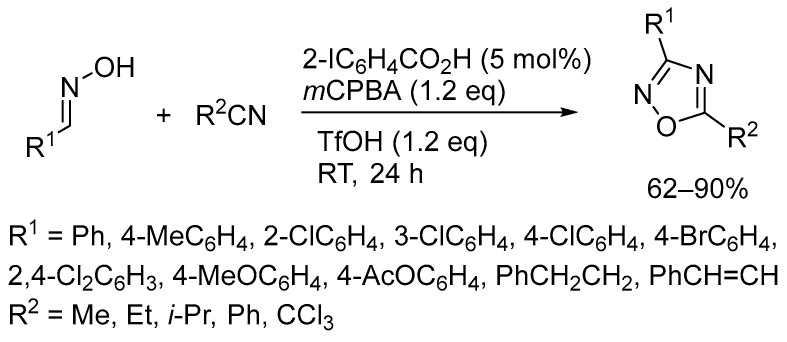
Oxidative cycloaddition of aldoximes with nitriles catalyzed by hypervalent iodine reagent.

Yoshimura, Zhdankin et al. described an oxidative cycloaddition reaction of aldoximes with nitriles promoted by 2-iodosylbenzoic acid triflate as an oxidant [130,131]. The authors demonstrated that the oxidant could be generated in situ from 2-iodobenzoic acid and *m*-chloroperoxybenzoic acid (*m*-CPBA) in the presence of TfOH. This fact allowed them to develop a synthetic protocol for the preparation of 1,2,4-oxadiazoles in the presence of catalytic amounts of 2-iodobenzoic acid and stoichiometric amounts of *m*-CPBA and TfOH (Scheme 28). The reaction proceeds at room temperature, affording corresponding 1,2,4-oxadiazoles in good yields (62–84%). Later, Wirth and colleagues reported [132] that this reaction can be carried out in a stoichiometric variant, using the presynthesized oxidant 2-iodosylbenzoic acid tosylate based on more accessible and cheap TsOH instead of TfOH. In this case, the reaction time can be reduced to 1 h. Unfortunately, the mechanism of this multistage reaction has not been studied by researchers, so the sequence of its stages remains unclear.

**Scheme 29:**
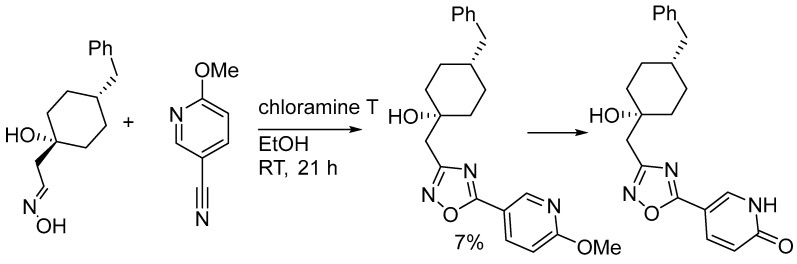
Synthesis of orally bioavailable cyclohexanol-based NR2B-selective NMDA receptor antagonists with analgesic activity.

Despite the fact that the oxidative cycloaddition of aldoximes with nitriles has not been described until recently, the method has already found application in medicinal chemistry. Anan and colleagues obtained orally bioavailable cyclohexanol-based NR2B-selective NMDA receptor antagonists with analgesic activity through the oxidative cycloaddition in a low yield with chloramine T as an oxidant (Scheme 29) [133].

**Scheme 30:**
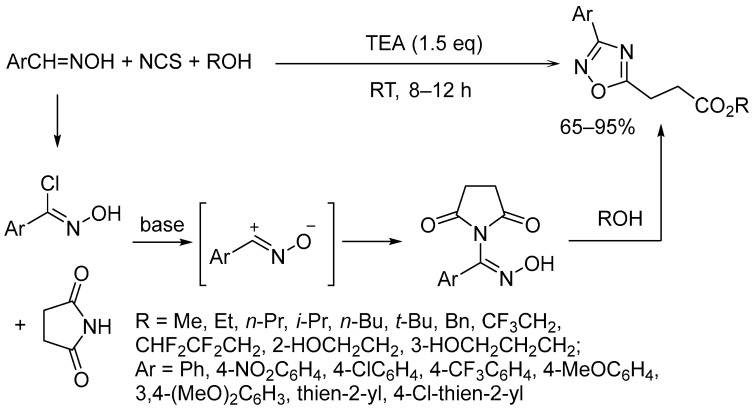
Synthesis of alkyl3-(3-aryl-1,2,4-oxadiazol-5-yl)propanoates (AAOPs) by reaction of aldoximes with NCS.

Indirectly, some light on how such a reaction can proceed is shed by Madabhushi et al. [134]. The authors used *N*-chlorosuccinimide (NCS) as an oxidant and the coupling partner for a base-catalyzed one-pot three-component synthesis of alkyl 3-(3-aryl-1,2,4-oxadiazol-5-yl)propanoates (AAOPs, the precursors for the preparation of CB2 agonists) from aryl aldoximes at RT (Scheme 30). The key intermediate is nitrile oxide, which is formed from aryl aldoxime under the reaction conditions.

**Scheme 31:**
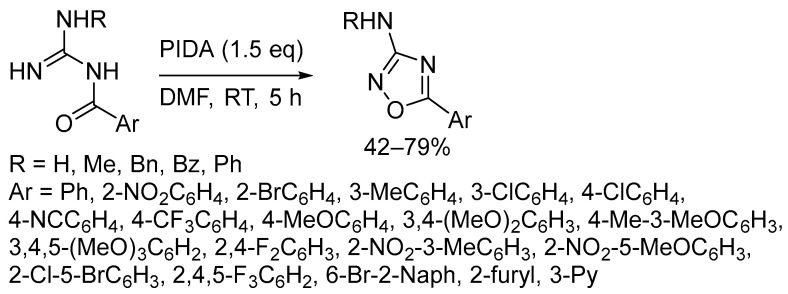
Synthesis of 3-amino-1,2,4-oxadiazoles from aromatic *N*-acylguanidines.

Among other papers devoted to oxidative cyclization affording 1,2,4-oxadiazoles, it is possible to distinguish two groups according to the type of bonds formed during coupling. Zhao, Gong et al. described an efficient and mild protocol for the synthesis of 3-amino-1,2,4-oxadiazoles in moderate to good yields by cyclizing aromatic *N*-acylguanidines with PhI(OAc)_2_ (PIDA) as an oxidant (Scheme 31) [135]. The process proceeds in DMF at room temperature for 5 h. 

**Scheme 32:**
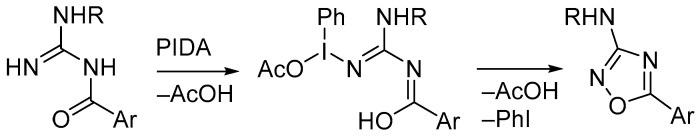
Mechanism of N–O oxadiazole bond oxidative formation with PIDA.

The reaction is an example of the N–O oxadiazole bond oxidative formation. The proposed mechanism based on the literature analogs and an *N*-iodinated intermediate involved are shown in Scheme 32.

**Scheme 33:**
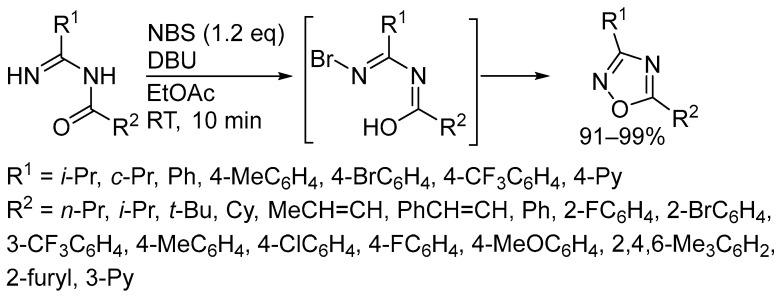
NBS-promoted oxidative cyclization of *N*-acyl amidines.

A similar reaction was reported by Yu, Chang, and colleagues for *N*-acyl amidines [136]. The authors describe an NBS-promoted oxidative cyclization of *N*-acyl amidines with ethyl acetate as the solvent at RT, which allows affording substituted 1,2,4-oxadiazoles in almost quantitative yields (91–99%) (Scheme 33). This process proceeds through the formation of *N*-bromination intermediate followed by the N–O bond formation as a result of dehydrobromination under basic conditions.

**Scheme 34:**
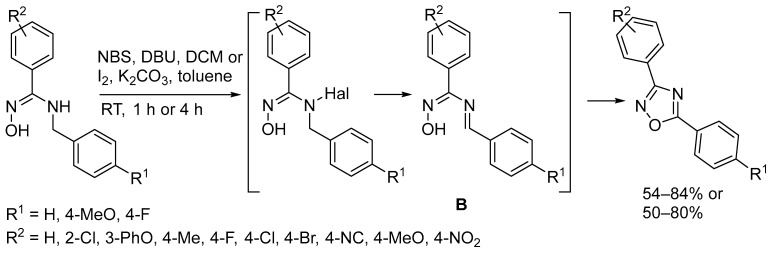
Oxidative cyclization of *N*-benzyl amidoximes.

Another group described the oxidative formation of the C–O bond. Vadagaonkar, Chaskar et al. reported the synthesis of 1,2,4-oxadiazoles from *N*-benzyl amidoximes by using such oxidizers as NBS or I_2_ in the presence of a base (Scheme 34) [137]. The NBS/DBU system achieves a few higher yields (54–84%) than the I_2_/K_2_CO_3_ system (50–80%). The reaction presumably occurs through *N*-halogenation of an amidoxime to form a halogenated derivative which in the presence of a base undergoes dehydrohalogenation and forms imine intermediate **B**. Intermediate **B** via cyclization-aromatization sequence offers 1,2,4-oxadiazole.

**Scheme 35:**
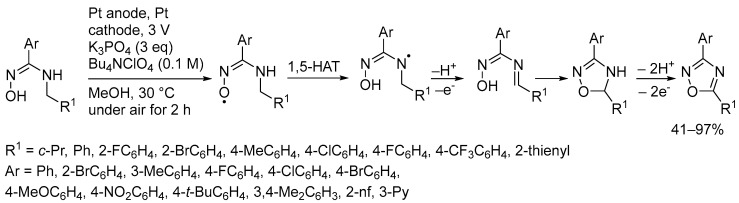
Cyclization of *N*-benzyl amidoximes by the electro-oxidation method.

Ye, Hu, and colleagues described an environmentally benign and sustainable approach for the synthesis of 3,5-disubstituted 1,2,4-oxadiazoles from *N*-benzyl amidoximes by the electro-oxidation method (Scheme 35) [138]. The transformation proceeds via the generation of the iminoxy radical through anodic oxidation, 1,5-hydrogen atom transfer (1,5-HAT), the second anodic oxidation, and cyclization/aromatization.

## 4. Miscellaneous

In addition to those mentioned above, several more scattered works on the low-temperature synthesis of 1,2,4-oxadiazoles by various methods have been published.

**Scheme 36:**
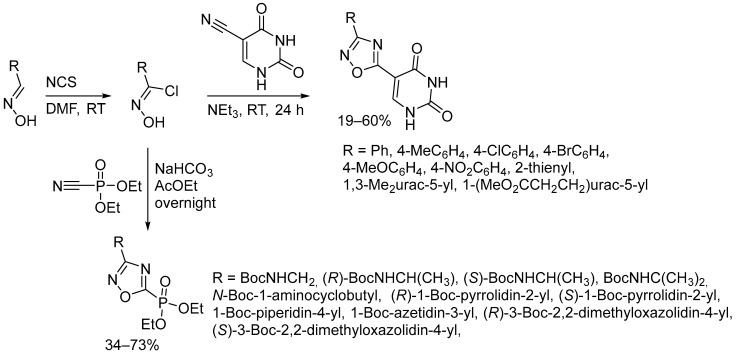
Synthesis of 5-(1,2,4-oxadiazol-5-yl)pyrimidine-2,4(1*H*,3H)-dienes and diethyl (1,2,4-oxadiazol-5-yl)phosphonates via 1,3-dipolar cycloadditions.

Jakubiec and Walczak reported a procedure for easy access to 5-substituted uracil derivatives, possessing the 1,2,4-oxadiazole ring on carbon C5 of the uracil ring [139]. The 1,3-dipolar cycloaddition reaction was applied for the construction of the heterocyclic ring (Scheme 36). The [2+3]-cycloaddition of nitrile oxides (formed in situ from the appropriate chlorinated 4-substituted benzaldoximes under basic conditions) with 5-cyanouracil as a dipolarophile gave the corresponding 5-(3-substituited-1,2,4-oxadiazol-5-yl)uracils in yields of 19–60% at RT. The similar 1,3-dipolar cycloaddition reaction was used by Grygorenko et al. for the preparation of 1,2,4-oxadiazole-substituted phosphonates (Scheme 36) [140].

**Scheme 37:**
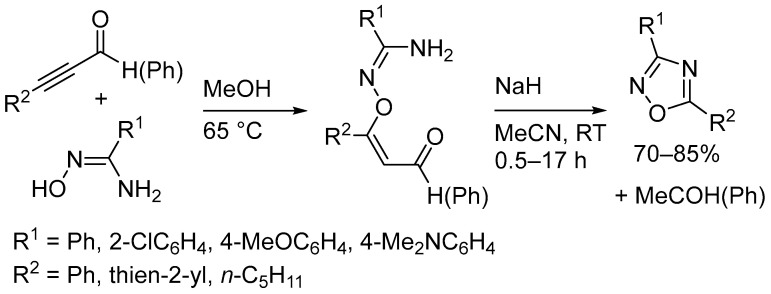
Synthesis of 1,2,4-oxadiazoles by cyclization of substituted *N*’-((3-oxoprop-1-en-1-yl)oxy)benzimidamides.

Kivrak and Zora described a synthesis of 1,2,4-oxadiazoles by cyclization of substituted *N’*-((3-oxoprop-1-en-1-yl)oxy)benzimidamides in the presence of NaH along with acetaldehyde or acetophenone as side products (Scheme 37) [141]. The reaction proceeds at RT, but heating is required to receive the starting compounds from amidoximes and *α*,*β*-alkynic aldehydes or ketones.

**Scheme 38:**
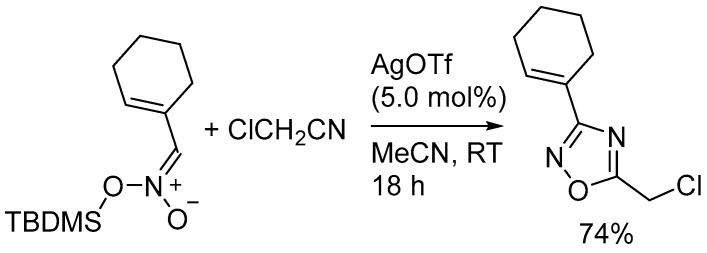
Metal-catalyzed [2+3]-cycloaddition of silyl nitronate and chloroacetonitrile.

Nikodemiaka and Koert found a novel pathway to 3,5-disubstituted 1,2,4-oxadiazoles via the metal-catalyzed [2+3]-cycloaddition of a substituted silyl nitronate (*t*-butyldimethylsilyl cyclohexenemethyleneazinate) and chloroacetonitrile (Scheme 38) [142]. The reaction is catalyzed by AgOTf. The silyl nitronate/nitrile cycloaddition step is followed by the elimination of TBSOH to deliver the 1,2,4-oxadiazole. The authors reported that the reaction proceeds at room temperature with a good yield (74%), but heating to 100 °C can fundamentally reduce the reaction time.

**Scheme 39:**
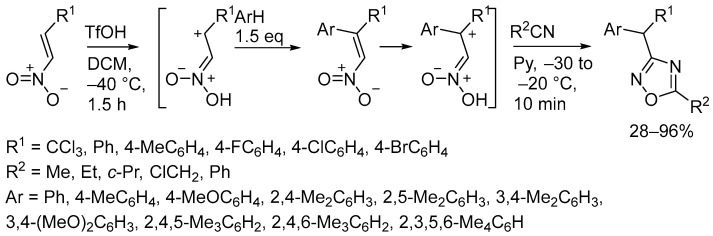
Synthesis of 3,5-disubstituted-1,2,4-oxadiazoles from nitroalkenes, arenes, and nitriles under the superelectrophilic activation.

Vasilyev and colleagues reported obtaining a wide range of 3,5-disubstituted-1,2,4-oxadiazoles in yields of 28–96% by tandem reaction of nitroalkenes with arenes and nitriles under the superelectrophilic activation conditions in TfOH (Scheme 39) [143]. The synthesis proceeds at diminished temperatures via generation of cations from various nitroalkenes under their reactions with arenes in TfOH and subject these species to consequent attack with nitriles, as *N*-nucleophiles.

**Scheme 40:**
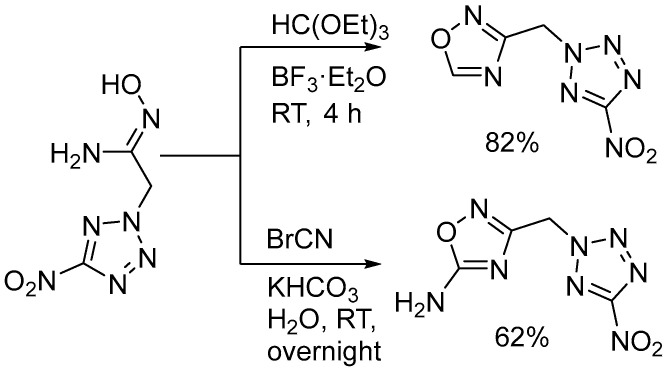
Synthesis of 3-(5-nitro-2*H*-tetrazol-2-yl)substituted-1,2,4-oxadiazoles.

Wang et al. found that 2-(5-nitro-2*H*-tetrazol-2-yl)acetamidoxime can serve as a starting compound for the preparation of the corresponding oxadiazoles at room temperature in good yields in both acidic and basic media (Scheme 40) [144]. The choice of conditions depends on which carboxyl derivative is the second reaction partner. The reaction with the orthoester is catalyzed by BF_3_, and with BrCN—by KHCO_3_.

**Scheme 41:**
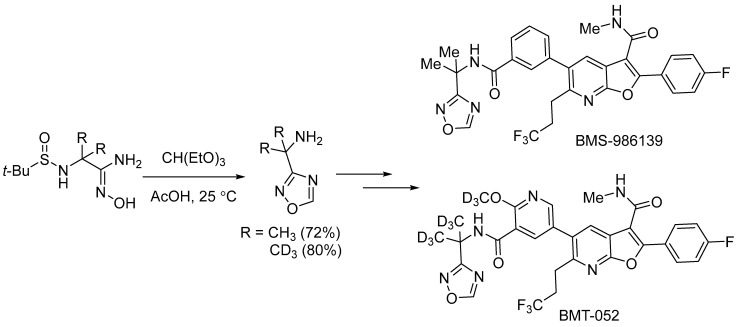
Synthesis of 2-(1,2,4-oxadiazol-3-yl)propan-2-amine and and its deuterated analog.

Another example of the room temperature condensation of amidoximes with orthoester is the synthesis of 2-(1,2,4-oxadiazol-3-yl)propan-2-amine and its deuterated analog in AcOH (Scheme 41) [145,146]. These compounds are employed in the preparation of pan-genotypic HCV NS5B polymerase inhibitors BMS-986139 and BMT-052.

**Scheme 42:**
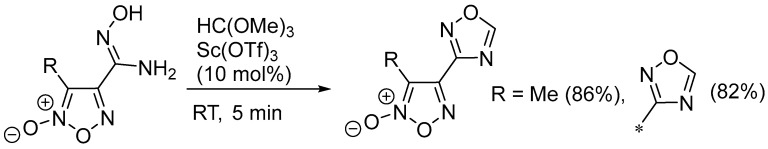
Sc(OTf)_3_-catalyzed condensation of amidoximes with orthoester.

The condensation of amidoximes with orthoester can also occur at room temperature under the catalysis of scandium triflate (Sc(OTf)_3_) (Scheme 42) [84]. The obtained compounds were evaluated as potential NO donors for cancer therapy [85].

**Scheme 43:**
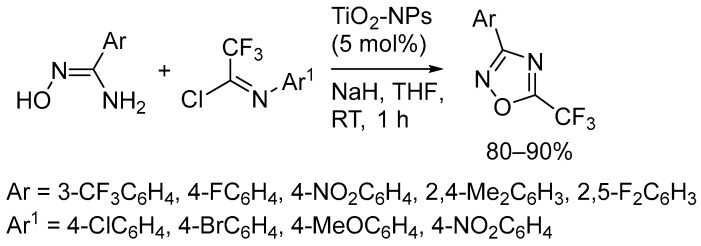
Synthesis of 3-aryl-5-(trifluoromethyl)-1,2,4-oxadiazoles.

Darehkordi and colleagues reported the synthesis of a new series of trifluoromethyl-1,2,4-oxadiazoles (Scheme 43) [147]. 3-Aryl-5-(trifluoromethyl)-1,2,4-oxadiazoles were synthesized by reaction of aryl amidoximes and trifluoroacetimidoyl chlorides in a one-pot manner in the presence of NaH and titanium dioxide nanoparticle as the catalyst.

**Scheme 44:**
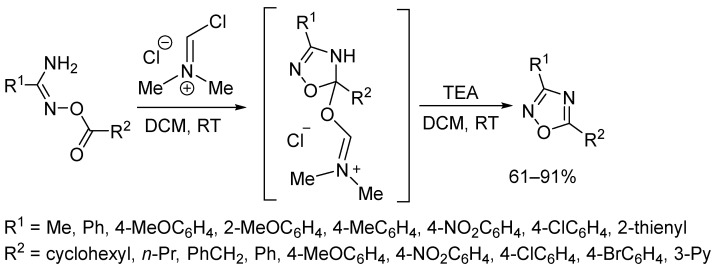
*O*-acylamidoxime cyclocondensation to 1,2,4-oxadiaozle using Vilsmeier reagent.

In 2018, Zarei [148] found that the Vilsmeier reagent activates both carboxylic acids for the *O*-acylation of amidoximes and *O*-acylamidoxime intermediates for the cyclocondensation into 1,2,4-oxadiazoles (Scheme 44). The reaction occurs in the presence of trimethylamine in CH_2_Cl_2_ at RT for 3 h. Moreover, based on this finding, the author developed the one-pot procedure for the synthesis of 1,2,4-oxadiazoles from nitriles and carboxylic acids in CH_2_Cl_2_ solution at RT. 

**Scheme 45:**
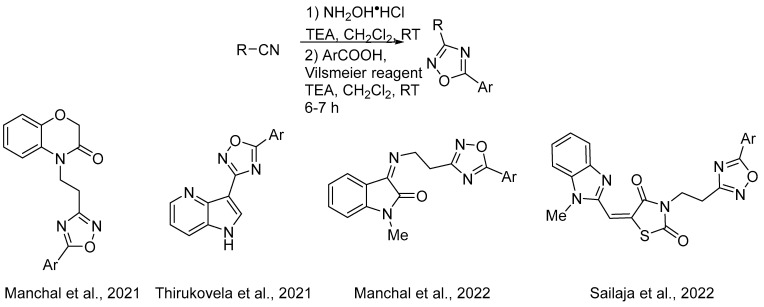
Synthesis of anticancer agents bearing 1,2,4-oxadiazole moiety using Vilsmeier reagent [149,150,151,152].

Later, Zarei’s method was applied to the synthesis of several new anticancer scaffolds (Scheme 45) [149,150,151,152].

## 5. Conclusions

The efficient synthesis of 3,5-disubstituted-1,2,4-oxadiazoles is an important goal in organic chemistry due to their wide application in drug discovery as bioisosteres of amide and ester functionalities. In the last two decades, the synthetic approaches towards 1,2,4-oxadiazole core have been significantly revised. Namely, novel methods that allow us to obtain these compounds at room temperature have been developed. This opens an avenue for the investigation of oxadiazoles with thermosensitive functions. Moreover, the new methods facilitate the use of the oxadiazole core as a linker in the design of pharmaceutically relevant molecules.

The methods discussed in this review are divided into three groups. The first of them are two-stage protocol requiring the preliminary preparation of *O*-acylamidoximes followed by cyclization under the action of organic bases (TBAF or TBAH). This approach emerged 20 years ago and has already won the recognition of researchers in the field of medicinal chemistry and material science. With its use, anticancer, antimicrobial, and antiviral agents, neuroprotectors, immunomodulators, and metabolic regulators were obtained. The said route exhibits noticeable efficiency in the complete cyclization of *O*-acylamidoximes to 1,2,4-oxadiazoles, including a wide range of heterocycles with various functional groups. The advantages of this method are swiftness, convenience, and simplicity of the process, and the reactions are carried out under mild conditions at 0 °C to room temperature. However, the use of *O*-acylamidoximes as starting materials is associated with the need for their preliminary preparation and isolation, which complicates the synthesis and reduces the overall yield of 1,2,4-oxadiazoles from amidoximes.

The second approach comprises the assembly of 1,2,4-oxadiazoles from amidoximes and various carboxyl derivatives or aldehydes in aprotic bipolar solvents (primarily DMSO) in the presence of inorganic bases. This approach profoundly simplifies the synthesis and increases the yield of oxadiazoles based on amidoximes. Methodologically, this pathway is close to the CuAAC reaction, ensuring the bridging of two molecular fragments into one molecule with an oxadiazole linker at mild conditions. Expectedly, this recently proposed approach found multiple applications in the design of bioactive molecules. The third group of methods includes diverse oxidative cyclizations. These reactions have not been widely used in drug design so far. We believe that the present review will stimulate interest and further advances in the synthesis and medicinal application of 1,2,4-oxadiazole-based small molecules. 

## Figures and Tables

**Table 1:** Bioactive 1,2,4-oxadiazoles synthesized using TBAF.

Activity	Scaffold	Ref.
Potential angiotensin II receptor antagonist (biological evaluation was not performed)	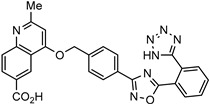	[54]
Inhibitors of Dengue virus nonstructural protein 5 (NS5) polymerase	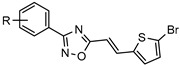	[55]
Antimalarial and antileishmanial compounds	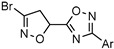	[56]
Catechol–O–methyltransferase (COMT) inhibitor marketed as anti-Parkinson drug (Opicapone)	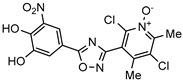	[57]
Antiretroviral agent targeting the HIV-1 nucleocapsid protein	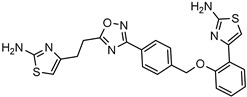	[58]
Inhibitor of serine hydroxymethyltransferase for the treatment of malarial	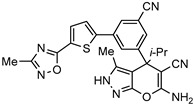	[59]
Fatty acid amide hydrolase (FAAH) inhibitors for the treatment of pain and CNS disorders	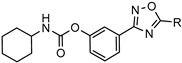	[60]
Kv7 channel opener for the treatment of epilepsy and pain	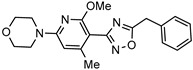	[61]
Antiproliferative action against malignant human adherent cell lines	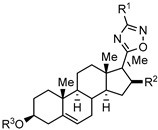	[62]
Antiproliferative action against malignant human adherent cell lines	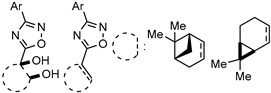	[63]
Hedgehog pathway inhibitors for the anticancer therapy	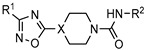	[64]
Dual akt and p70S6 kinases inhibitor for the anticancer therapy (solid tumors)	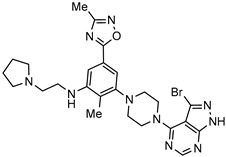	[65]
Bombesin receptor subtype-3 agonist for the obesity treatment	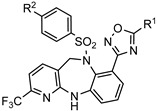	[66]
Melanocortin-4 receptor (MC4R) agonist for the obesity treatment	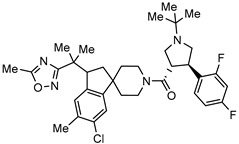	[67]
Sphingosine kinase 1 inhibitors for modulating the production of sphingosine-1-phosphate (S1P)—a potential treatment of cancer, sickle cell disease, atherosclerosis, asthma, diabetes, and fibrosis	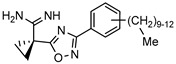	[68]
Sphingosine kinase 2 inhibitors for modulating the production of sphingosine-1-phosphate (S1P)—a potential treatment of cancer, sickle cell disease, atherosclerosis, asthma, diabetes, and fibrosis	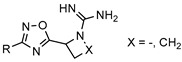	[69]
Sphingosine-1-phosphate receptor-1 (S1PR_1_) agonists for the therapy of autoimmune diseases (preliminary, relapsing, remitting multiple sclerosis) and the prevention of transplant rejections	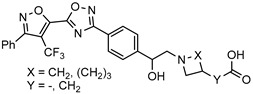	[70]
	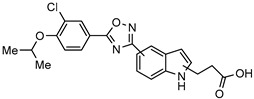	[71]
	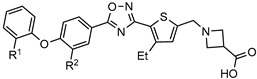	[72]
Inhibitor of penicillin binding proteins with antibacterial activity against Gram-positive strains	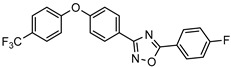	[73]
Neuronal cells protectors preventing the oxidative stress-induced cell death of glutamate-challenged HT22 hippocampal neurons	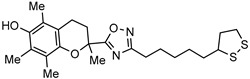	[74]
	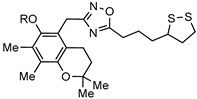	
	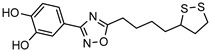	[47]
	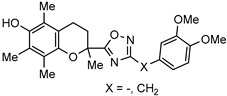	[75]
	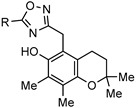	
Serotonin 5-HT1A and dopamine D2 dual-receptor agonist for the treatment of Parkinson’s diseases	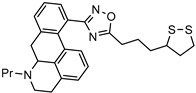	[76]
Acyl CoA:diacylglycerol acyltransferase (DGAT1) inhibitors for the therapy of obesity and obesity-induced diabetes	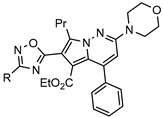	[77]
Agents for the treatment *Toxoplasma gondii* infections	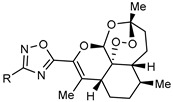	[78]
Negative allosteric modulators (NAMs) of metabotropic glutamate receptor 5 (mGluR5) for neurodegenerative diseases treatment	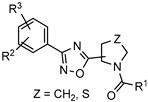	[79]

## Data Availability

Not applicable.
